# The Effects of Heat Straightening Temperature on the Microstructure and Properties of 7N01 Aluminum Alloy

**DOI:** 10.3390/ma12182949

**Published:** 2019-09-11

**Authors:** Shuai Li, Honggang Dong, Lei Shi, Xingxing Wang, Zhongying Liu, Linjian Shangguan, Yusong Tian

**Affiliations:** 1 School of Mechanical Engineering, North China University of Water Resources and Electric Power, Zhengzhou 450045, China; lyctlishuai@163.com (S.L.); paperwxx@126.com (X.W.); liuzhongying87@126.com (Z.L.); sgljbh@163.com (L.S.); 2 New Energy Materials and Technology Institute Ltd. of Dalian University of Technology, Qingdao 266200, China; 3 School of Materials Science and Engineering, Dalian University of Technology, Dalian 116024, China; sllweld@mail.dlut.edu.cn; 4 School of Electronic Engineering, Xidian University, Xi’an 710126, China; imtianyusong@163.com

**Keywords:** heat straightening, mechanical properties, corrosion behavior, natural aging

## Abstract

The 7N01 aluminum alloys are always used in vehicles, but heat treatment can deteriorate mechanical properties and corrosion resistance, which limits its utilization. In this paper, the influences of the temperature of heat straightening on the corrosion behavior and mechanical properties of 7N01 aluminum alloy are investigated. Most of the initial Guinier Preston (GP) zones dissolve into the matrix during heat treatment process, while the grain boundary precipitates have no obvious change. The precipitates of the samples after heat treatment mainly consist of high density GP zones due to the natural aging effect, which result in the recovery of micro-hardness. Although heat treatment decreases the mechanical properties of 7N01 aluminum alloy, there is no obvious difference in mechanical properties of the specimens after different heat treatment conditions. The corrosion resistance of heat treatment samples decreases significantly compared with the base metal, which is attributed to enhancing the difference between the potential of the alloy matrix and the grain boundary.

## 1. Introduction

Al–Zn–Mg alloy is a typical aging strengthening, medium-strength aluminum alloy, which is widely applied in high-speed railway, automobile and other transportation vehicle structural parts, because of its acceptable specific strength, good weldability and corrosion resistance [1,2]. There are lots of methods to connect the aluminum alloy, while welding is widely applied, owing to its advantages of simple operation and low cost, especially the metal inert-gas (MIG) arc welding [3,4]. The aluminum alloys will undergo thermal cycling during the welding process, then the welding deformation occurs. The welding deformation is inevitable for the welded structural parts due to the thermal expansion and contraction effect of metal parts, especially for the aluminum alloy, which always affect the dimensional accuracy and appearance of weldment. Consequently, some measures are often taken to control welding deformation, such as post-weld correction.

Generally speaking, the post-weld correction consists of mechanical straightening and heat straightening [5,6]. The mechanical correction method mainly refers to the use of external force to promote the deformation of the structural parts in the opposite direction of the welding deformation; then the new mechanical correction deformation offsets the welding deformation. Basically, large-tonnage presses or flange straightening machines are often used for batch correction in the industry and the mechanical correction has the advantages of high efficiency and low cost. The heat straightening can be divided into two types: integral heating and local heating. The integral heating can be used when the welding deformation is large. However, the disadvantage of this method is that the whole heating after welding tends to produce metallurgical side effects. Local heating is often performed by oxy-acetylene flame. The welding deformation zone is heated by the moving oxy-acetylene flame and then quenched with water immediately. Since heating makes metallic materials expand and cold makes them contract, in the high temperature places, the thermal expansion of the material is restricted by the rigidity of the structural member, resulting in local compression deformation [5,6]. Then, the new contractive plastic deformation counteracts the former welding deformation. Thanks to the advantages of simple equipment and flexibility, the local flame correction is always applied in post-weld structural parts.

Lots of investigations have pointed out that the mechanical properties and corrosion behavior of Al–Zn–Mg(Cu) alloy are mainly related to the heat treatment process [1,7,8]. The heat straightening will induce the transformation of microstructure and properties of aluminum alloy. At present, the investigations of heat straightening mainly focus on heat straightening temperature, the number of heat straightenings, and quenching process during heat straightening. Jiang et al. [9] and Lu et at. [10] used a direct oxy-acetylene flame and investigated the corrosion susceptibility and mechanical properties of Al–Zn–Mg alloys under different heat correction temperatures. The main problem of this experimental method is that the thermal correction process cannot be controlled accurately, because the heat straightening process is mainly based on the workers’ experience and has less repeatability than is desirable. Li et al. [1] and Dong et al. [11] investigated the number of heat straightenings on the corrosion susceptibility and mechanical properties of Al–Zn–Mg alloy, based on the method of heat treatment simulation, and pointed out that the corrosion resistance decreased with an increase of the number of heat straightenings. Li et al. [12] investigated the influence of the quenching process during flame correction on the microstructure and performances of Al–Zn–Mg alloy and revealed that the Al–Zn–Mg alloy has the lowest corrosion resistance when the air quenching process is conducted. The specimens got better corrosion resistance when treated with air cooling for 5 min, followed by the water quenching process. In this paper, the heat straightening process was simulated through non-isothermal heat treatment (NIHT) process. The aim was to investigate the evolution of microstructure and corrosion behavior under the actual heat straightening temperature of 300 °C and 350 °C and provide a basis for optimizing heat straightening parameters.

## 2. Experimental Procedure

### 2.1. Heat Treatment Process

**Figure 1:**
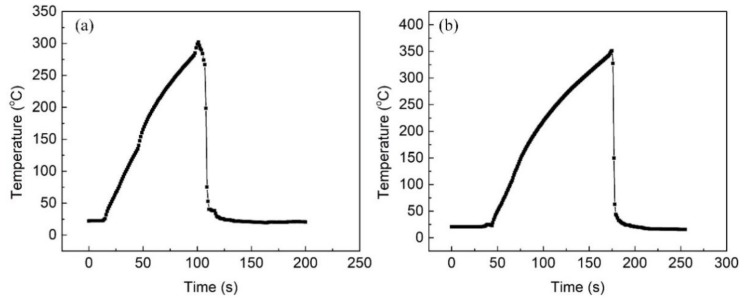
The thermal cycle curves of 7N01 aluminum alloy under water quenching after (**a**) 300 °C and (**b**) 350 °C heat treatment.

The 7N01 aluminum alloy specimens in T4 condition with the size of 150 × 40 × 5 mm^3^ were prepared, and the chemical composition of the alloy is listed in Table 1. The T4 state related to solution heat treatment at the temperature of 470 °C for 1 h, followed by quenching in water, and finally natural aging. The non-isothermal heat treatment process was performed through air furnaces (KSL-1200X, HeFei KeJing Materials Technology Co., LTD, Hefei, China) and a temperature recorder (Yokogawa GP10, Yokogawa Electric Co., Musashino, Japan). The thermal cycle curves during the heat treatment process was recorded by a K-thermocouple embedded in the geometric center of the samples. The specimens with an embedded K-thermocouple were dealt with in the air furnace, then taken out and quenched in water immediately when the peak temperature reached the preset values. The preset temperatures were 300 °C and 350 °C; the corresponding heating times were set to 90 s and 120 s according to actual process, respectively. The typical thermal cycle curves are shown in Figure 1. It is noted that all experiment were performed on the 45th day after the heat treatment process, considering natural aging [10].

### 2.2. Mechanical Property Experiments

**Figure 2:**
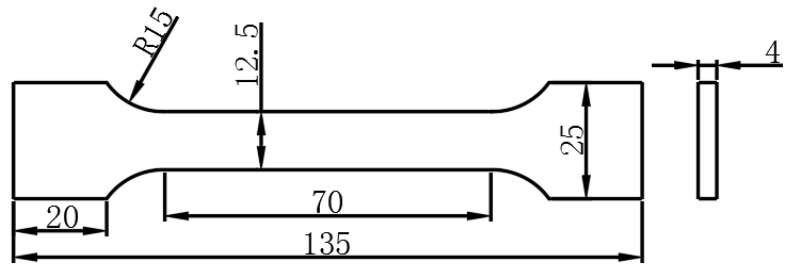
Sample for mechanical property testing (mm).

Figure 2 shows the sizes of the tensile specimens and the tensile test was performed on the DNS100 (Changchun Testing Machine Institute Co., Ltd., Changchun, China). The tensile speed was 5 mm/min during tensile test. The micro-hardness test was carried out with an MVC-1000B (Shanghai Jimin Testing Instrument Co., Ltd., Shanghai, China) apparatus with a load of 100 g for 15 s.

### 2.3. Corrosion Experiment

The intergranular corrosion (IGC) test was carried out according to standard ASTM G110-92 with the mixed corrosion solution of 1.0 M NaCl + 0.01 M H_2_O_2_ [13]. The dimensions of the IGC specimens were 40 × 25 × 5 mm^3^ and the experiment was conducted in a water bath with the DK-98-II device (Tianjin TaiSiTe Instrument Co., Ltd., Tianjin, China) at the temperature of 35 ± 2 °C for 12 h. The exfoliation corrosion test was carried out in the solution of 4 M NaCl + 0.5 M KNO_3_ + 0.1 M HNO_3_ with the sample sizes of 40 × 30 × 5 mm^3^ depending on the standard ASTM G34-01 [14].

The potentiodynamic polarization tests were carried out in an electrochemical workstation of type CS350 (Wuhan Corrtest Instruments Corp., Ltd., Wuhan, China). Electrochemical experiments were performed by using a typical three-electrode test system; namely, an Ag/AgCl electrode as a reference electrode, a large platinum sheet as a counter electrode, and the studied alloy as the working electrode. The range of the potentiodynamic polarization tests was ±200 mV (relative to open circuit potential) with the scan speed of 1 mV/s. In order to keep the potential stable, the open circuit potential test was conducted for 30 min before the polarization curve test. All experiments were performed at room temperature with the solution of 1.0 M NaCl, and the exposed area of the samples was 0.95 cm^2^.

### 2.4. Microstructural Investigations

After grinding and polishing, the metallographic samples were put into the mixed solution—HF:HCl:HNO_3_:H_2_O = 1:1.5:2.5:95. The optical metallographic photos of heat treatment samples were captured with a Leica MEF4 optical microscope (OM, Wetzlar, Germany). The fracture and corrosion morphology were observed by ZEISS SUPRA 55 field emission scanning electron microscopy (SEM, Oberkochen, Germany). The heat treatment samples were prepared for transmission electron microscope (TEM) observation. First, the samples were polished carefully to around 30 μm in thickness by SiC sandpaper, and stamped to 3 mm in diameter. Afterwards, the thin foils were handled by twin-jet electropolishing with the voltage of 20 V at the temperature of −30 °C, and the solution was prepared by 30% HNO_3_ and 70% CH_3_OH. The evolution of precipitation in the alloy after heat treatment process was observed through Tecnai G^2^20s-twin TEM (Waltham, MA, USA) with the operating voltage of 200 kV.

### 2.5. Differential Scanning Calorimetry (DSC) Analysis

The evolution of precipitation of 7N01 aluminum alloys with different heat treatment conditions were analyzed by a TA Q20 differential scanning calorimeter (DSC, New Castle, DE, USA) and the weight of a DSC sample was about 20 mg. The temperature range was set as 25–500 °C at the heating rate of 10 °C/min, with pure aluminum as reference sample.

## 3. Results

### 3.1. Mechanical Properties

**Figure 3:**
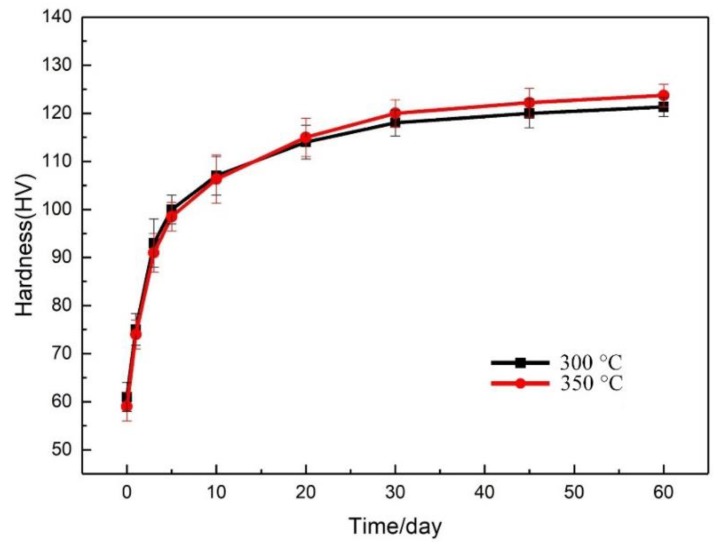
The evolution of micro-hardness in samples during natural aging.

The hardness evolution of 7N01 aluminum alloy after heat treatment is shown in Figure 3. It is obvious that the natural aging phenomenon occurred for the heat-treated 7N01 aluminum alloy, judging from the evolution of hardness. The hardness curve can be divided into three stages: the hardness increases rapidly in the first 5 days of natural aging, then gradually slows down from 5 days to 30 days, and finally, tends to be stable after 45 days. The hardness of the sample treated at 350 °C is slightly higher than that of the specimen treated at 300 °C after stabilization. The natural aging effect nearly disappears after 45 days, so all the experiments in the paper were carried out after 45 days of natural aging.

**Figure 4:**
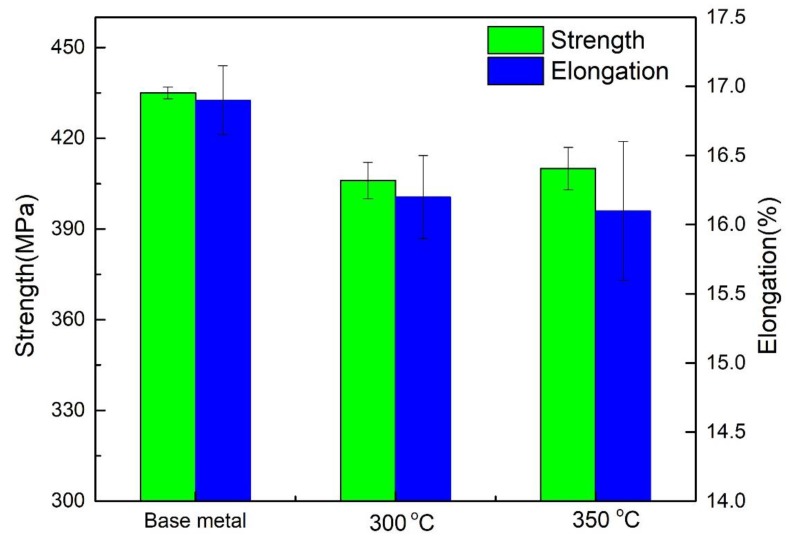
The mechanical properties of 7N01 aluminum alloy with different thermal cycling conditions.

Figure 4 shows the tensile properties of 7N01 aluminum alloy under different heat treatment peak temperatures. For the base metal, the tensile strength and elongation rate are 435 MPa and 16.9%, respectively. The tensile strength and elongation rate decrease after the heat treatment process compared with the base metal. After heat treatment at the peak temperature of 300 °C, the tensile strength of the 7N01 aluminum alloy reaches 406 MPa, which is slightly lower than 410 MPa obtained at the peak temperature of 350 °C. Additionally, the heat treatment peak temperature has nearly no influence on the elongation.

**Figure 5:**
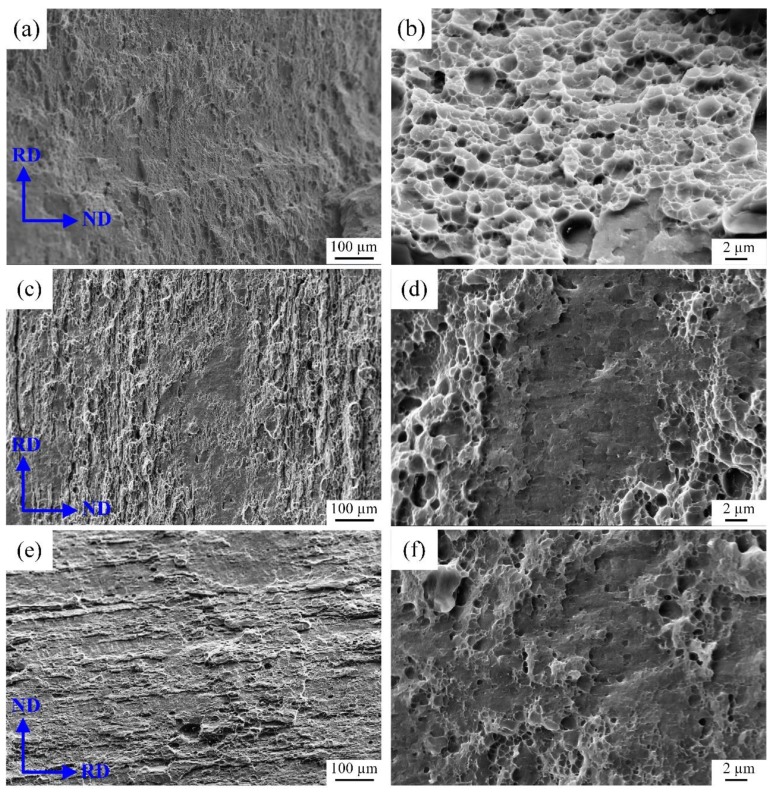
The fracture morphology and corresponding magnified images of 7N01 aluminum alloy (**a**,**b**) base metal; (**c**,**d**) 300 °C and (**e**,**f**) 350 °C.

The fracture surface morphology of the samples after different heat treatments are displayed in Figure 5. The fracture surface of the base metal exhibits a relatively uniform small dimples, which is dominated by ductile fracture, as shown in Figure 5a,b. For the heat treatment samples, the local areas of fracture morphology display smooth planar features at a low magnification and a lamellar tearing characteristics appears at the edge of plane. It also can be seen that very small and shallow dimples exist in local areas of the plane in high magnification, as shown in Figure 5c–f. Consequently, the tensile specimens after heat treatment exhibit mixed fracture characteristics.

### 3.2. Corrosion Behavior

**Figure 6:**
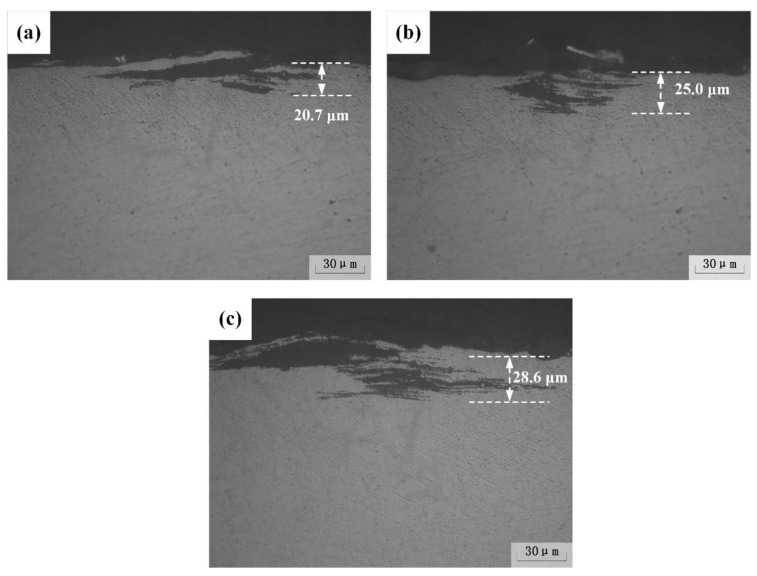
The cross-sectional view of (**a**) base metal, the treated samples under (**b**) 300 °C and (**c**) 350 °C after intergranular corrosion.

The immersion corrosion experiment of the specimens after heat treatment with different peak temperatures was carried out according to the standard of ASTM G110-92, and the results are shown in Figure 6. The corrosion morphology of all samples is dominated by pitting corrosion and exfoliation corrosion. There is no typical network-like intergranular corrosion characteristic, because grain boundary precipitates discontinuously distribute.

**Figure 7:**
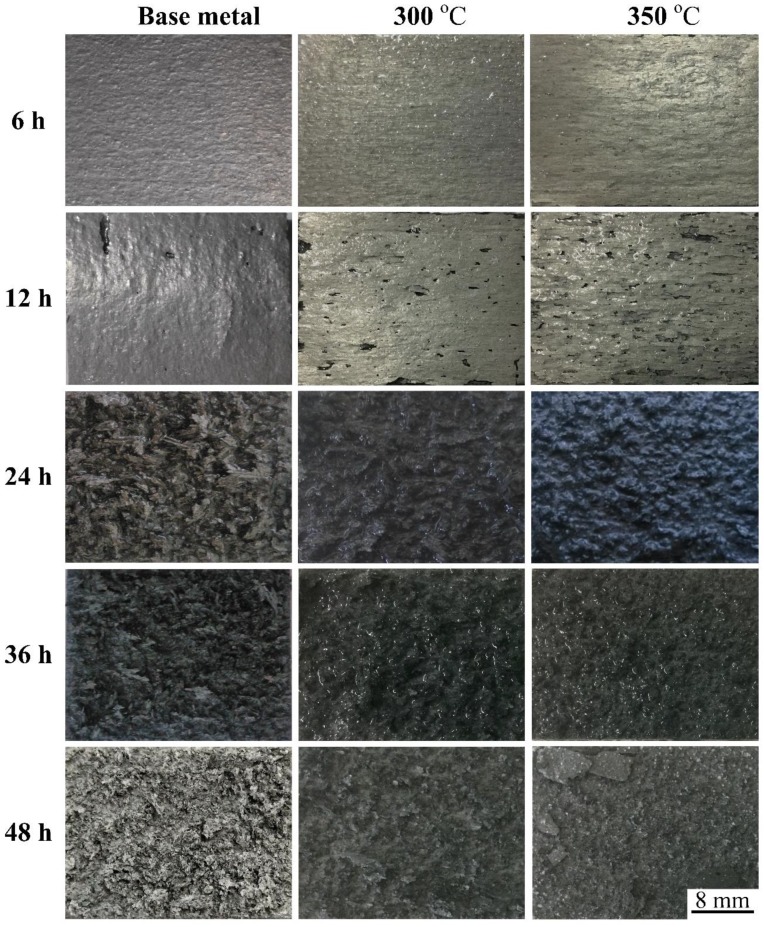
Surface evolution of all samples after exfoliation corrosion at different time periods.

The evolution of the exfoliation corrosion morphology of specimens with different thermal cycling conditions is displayed in Figure 7. In the early stage of exfoliation corrosion, the surface of the 7N01 aluminum alloy loses its metallic luster gradually; then corrosion types develop from pitting corrosion to more serious overall corrosion gradually. The exfoliation corrosion resistances of specimens after thermal cycling are worse than that of base metal, and the sample after heat treatment with the peak temperature of 350 °C shows the worst exfoliation corrosion resistance among the three samples, when the immersion time is less than 12 h. Moreover, for the heat treatment samples, the metal layer lift-up phenomenon occurs when the immersion time is 12 h, while the same phenomenon appears for the base metal in the period between 12 h and 24 h. Consequently, in the early stage of actual exfoliation corrosion tests, the exfoliation corrosion resistance of 7N01 aluminum alloy is ranked in the following order: base metal > 300 °C > 350 °C. All the specimens display overall corrosion features and the different of exfoliation corrosion morphology is not obvious when the immersion time is more than 12 h. The detailed variation of the exfoliation corrosion grade of 7N01 aluminum alloy with the immersion time is listed in Table 2.

**Figure 8:**
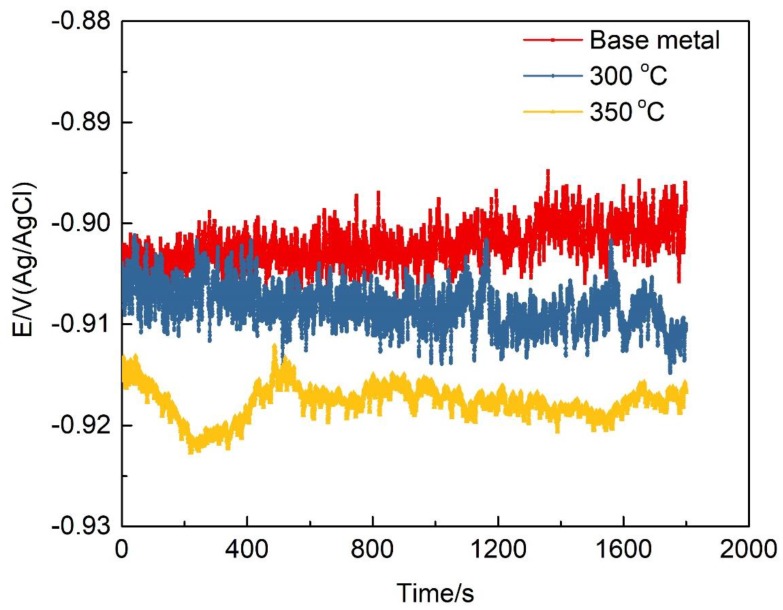
The open circuit potential of 7N01 aluminum alloy in a 1 M NaCl aqueous solution under different heat treatment conditions.

**Figure 9:**
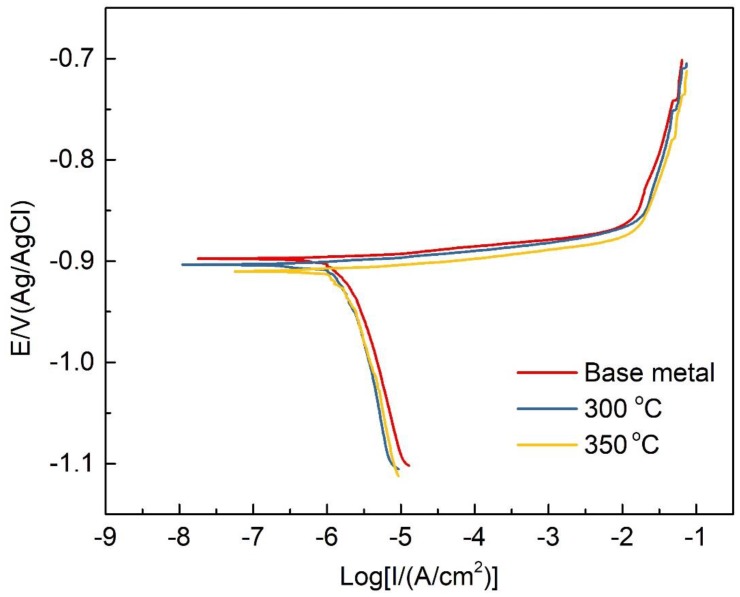
Potential dynamic polarization curves of 7N01 aluminum alloy in a 1 M NaCl aqueous solution under different heat treatment conditions.

The open circuit potentials of samples in Figure 8 with different heat treatment conditions was tested with the solution of 1 M NaCl. It can be seen that the open-circuit potential reaches a relatively stable state at around 30 min. The relevant investigations revealed that the difference of open-circuit potential is mainly determined by the distribution of grain boundary precipitates. Figure 9 shows the typical polarization curves of the specimens after different heat treatment process and the related parameters are listed in Table 3. The anodic and cathode cures were considered and the Tafel-type fit of the data was obtained to estimate the corrosion potential (*E*_corr_) and corrosion current density (*i*_corr_) from the polarization curves. The corrosion potential is an electrochemical thermodynamic parameter, which reflects the difficulty of corrosion. The more negative the corrosion potential, the greater the tendency of the alloy to be corroded [15]. The corrosion current density is an electrochemical kinetic parameter, indicating the rate of corrosion. The higher the value, the faster the corrosion proceeds. The electrochemical parameters in Table 3 indicate that the corrosion tendency of the 7N01 aluminum alloy improves after heat treatment, and the corrosion potential of the sample after heat treatment at the temperature of 350 °C is the lowest. Meanwhile the corrosion current density and corrosion rate of samples with different heat treatment conditions have an opposite change trend with the corrosion potential. The main reason lies in that the fitting zones from the polarization curves are subjective. It is also noted that the difference of corrosion current density and corrosion rate of the samples with different thermal cycling conditions is small.

**Figure 10:**
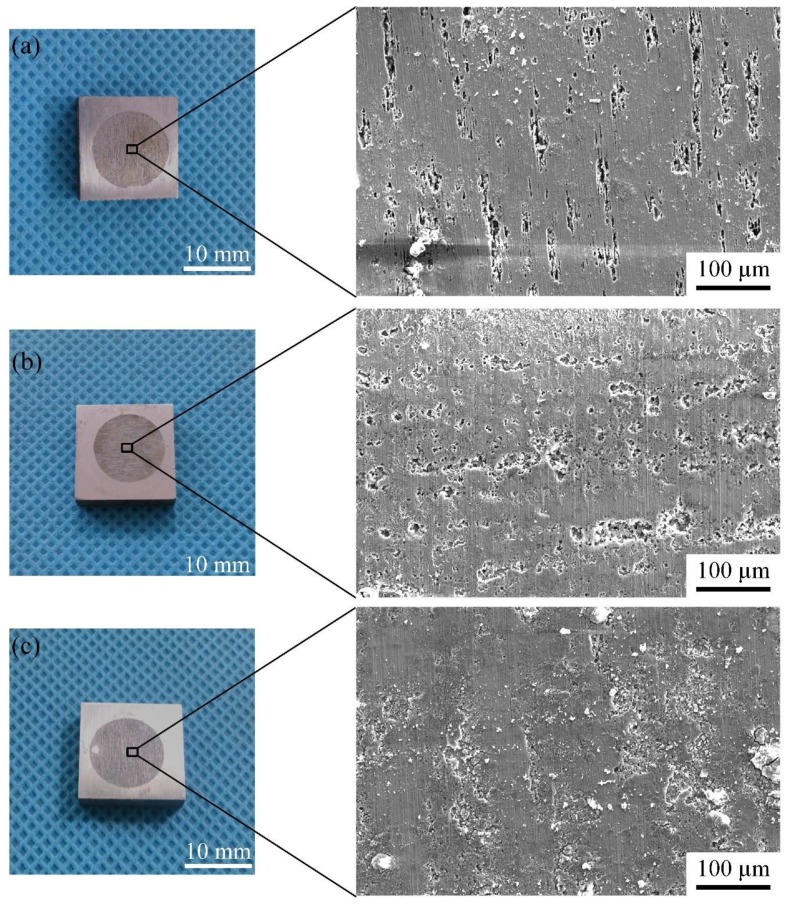
Macro morphology and the corresponding SEM images of the sample with different heat treatment conditions after dynamic potential polarization test. (**a**) Base metal; (**b**) 300 °C and (**c**) 350 °C.

The macro morphology and the corresponding SEM images of the samples with different heat treatment conditions after polarized testing are shown in Figure 10. It can be seen that the surface of the polarized area has a relatively uniform corrosion morphology, and the macroscopic image has no significant difference. A large number of narrow and small corrosion grooves are randomly distributed on the surface of the base metal through SEM observation; and the corrosion grooves on the surface of samples after heat treatment under 300 °C become wider and longer, and the corresponding area fraction of corrosion grooves become larger. For the sample after heat treatment with the peak temperature of 350 °C, the long strips of corrosion pits gathered together and further developed into a larger area of corrosion pits, and the characteristics of a single strip-shaped groove are not obvious, which indicats more serious corrosion than those of the other two specimens.

### 3.3. Microstructure Analysis

**Figure 11:**
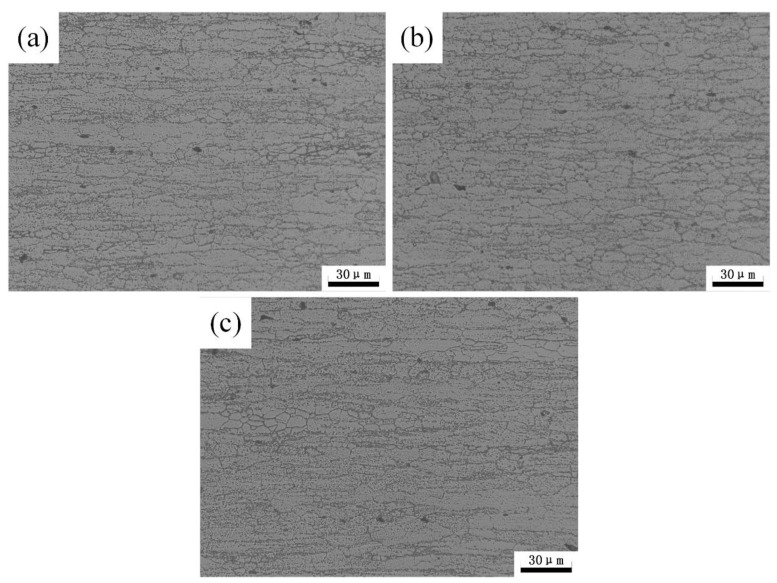
Metallographic structure of 7N01 aluminum alloy for (**a**) base metal; (**b**) 300 °C and (**c**) 350 °C.

The typical optical metallurgical images of specimens after different heat treatment conditions are shown in Figure 11. The base metal is featured with typical rolling microstructure, which consists of deformed grains elongated in the rolling direction and fine recrystallized grains. There is no obvious change of the optical metallurgical images of the samples after heat treatment, which is likely attributed to the recrystallization in 7N01 aluminum alloy.

**Figure 12:**
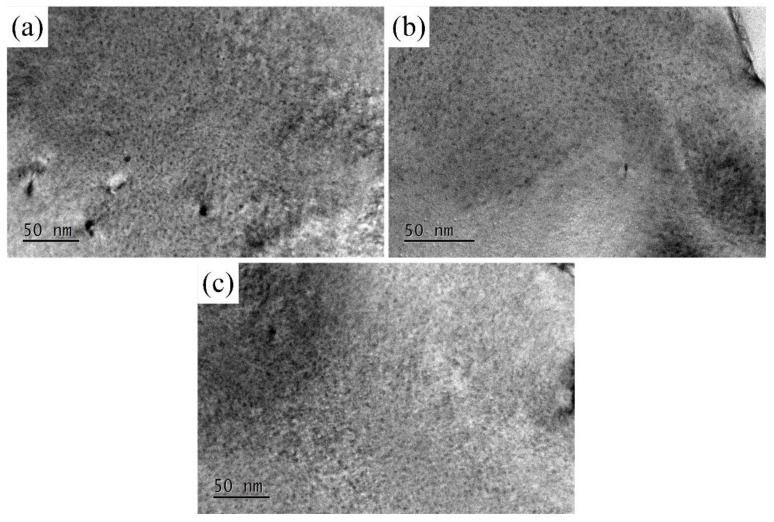
TEM images of matrix precipitate morphology in 7N01 aluminum alloy after different heat treatment conditions. (**a**) Base metal; (**b**) 300 °C and (**c**) 350 °C.

The 7N01 aluminum alloy in T4 state mainly consists of natural aging clusters formed by the aggregation of solute atoms of the aluminum matrix. The strength effect of 7N01 aluminum alloy in the T4 state is mainly associated with natural aging clusters; namely, GP zones. The distribution of matrix precipitates of 7N01 aluminum alloy are shown in Figure 12. During the precipitation of 7N01 aluminum alloys, a GP zone forms from 20 to 120 °C, an η′ phase is generated from 120 to 250 °C, and an η phase develops during 150–300 °C [2,3]. It can be seen all the samples consist of the high-density disc-shaped GP zones with the size of about 1 nm, which is mainly attributed to the formation temperature range of GP zones and the initial state of 7N01 aluminum alloy, as shown in Figure 12a–c.

**Figure 13:**
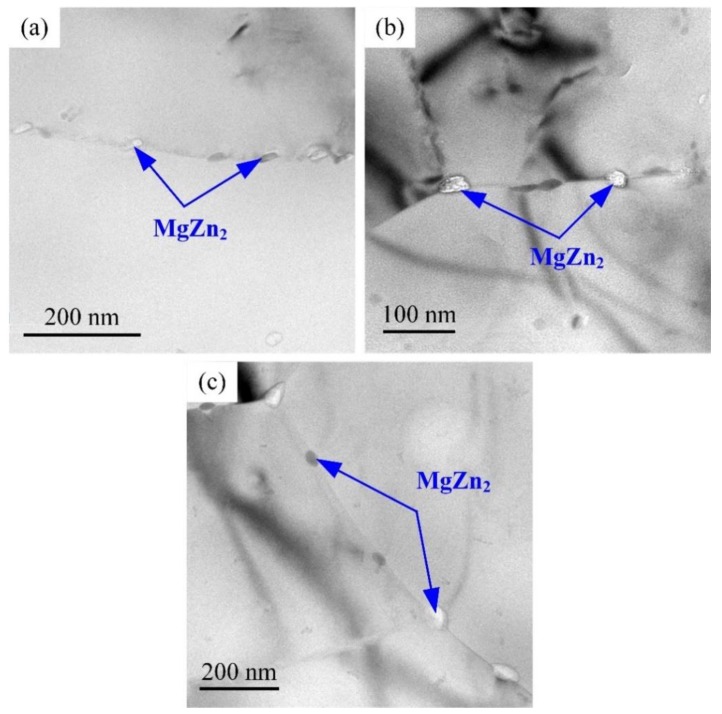
TEM images of grain boundary morphology in 7N01 aluminum alloy after different heat treatment conditions. (**a**) Base metal; (**b**) 300 °C and (**c**) 350 °C.

Figure 13 shows the evolution of grain boundary precipitates of the samples with different heat treatment conditions. It can be seen that grain boundary precipitates MgZn_2_ are discontinuous [3]; the size of precipitates is about 35 nm and no obvious grain boundary-free precipitation zone is observed, as displayed in Figure 13a. In addition, the distribution morphology of the grain boundary precipitates MgZn_2_ has no obvious change after heat treatment with a size of about 50 nm, as shown in Figure 13b,c.

### 3.4. DSC Curve Analysis

**Figure 14:**
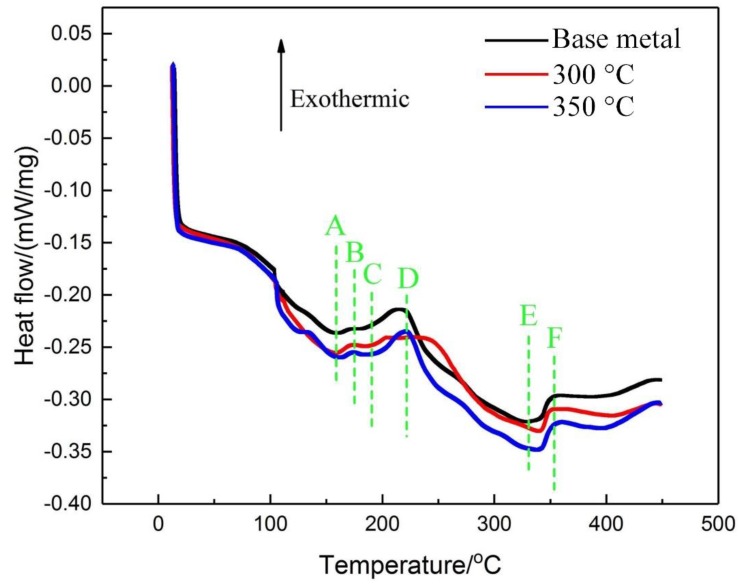
DSC curves of 7N01 aluminum alloy under different thermal cycling conditions.

The DSC curves of 7N01 aluminum alloy under different heat treatment conditions are shown in Figure 14. It can be seen that the features of the curves of the base metal and heat treatment samples are similar, all showing obvious three endothermic peaks (A, C, and E) and three exothermic peaks (B, D, and F). The peak A with the temperature range of 100–160 °C implies the dissolution of the GP zones; the smaller peak B (165–180 °C) is related to the formation of the η’ sub-stable phases; and the peak C (180–210 °C) indicates partial dissolution of the precipitates. The appearance of peak D is related to the formation of η phases. In addition, it is noted that the difference of the characteristics of peak D is obvious, and the temperature range of peak D of the sample with heat treatment’s peak temperature of 300 °C is larger. The peak E implies the dissolution of η phases and the appearance of peak F is related to the high temperature precipitates. The relevant investigations have shown that the features of DSC curves are related to many factors, including the size, volume fraction, and type of precipitates in the initial state of the sample, and the dissolution and precipitation temperature range of the GP, η, and η phases are coincident [16]. Additionally, in some temperature ranges, different thermal reactions of multiple second phases may occur simultaneously.

## 4. Discussion

The evolution of the microstructure for the Al–Zn–Mg alloy during non-isothermal heat treatment is different compared with the traditional isothermal heat treatment. In the non-isothermal heat treatment process, the precipitation, growth, and re-precipitation of the precipitates are involved. Nicolas and Deschamps [17] studied the precipitation kinetics of Al–Zn–Mg alloy in T6 state during non-isothermal heating systematically. They found out that most of the η’ phase dissolves into the matrix during non-isothermal heat treatment. Considering the heating rate, peak temperature used in the experiment, and the poor thermal stability of the GP zones, it is concluded that the evolution of the microstructure of the base metal is mainly consists of the dissolution of GP zones; namely, most of GP zones dissolve into the matrix, and result in the decrease of hardness, greatly. After heat treatment, the supersaturated solid solution is formed; then, the GP zone is re-formed from the matrix during natural aging. Consequently, the hardness of the heat treatment samples increases gradually with time until it reaches a steady state. The driving force of atomic segregation during natural aging is large because the supersaturated solid solubility of the sample quenched from 350 °C to room temperature is greater than that of the sample quenched from 300 °C to room temperature. Consequently, the volume fraction and size of the GP zone will also increase correspondingly for the sample after heat treatment with the peak temperature of 350 °C. The hardness and tensile strength of the sample after heat treatment at the peak temperature of 350 °C are slightly greater than those of the sample after heat treatment at the peak temperature of 300 °C. The dissolution temperature of stable MgZn_2_ phase is around 340 °C [16]. In addition, the duration of the 7N01 aluminum alloy being exposed to 340 °C or more for a very short time (a few seconds), and the MgZn_2_ phase, are too late to dissolve and diffuse. Consequently, the grain boundary precipitates of the alloy did not change significantly at the two peak temperatures.

The grain boundary precipitates become bigger and more discontinuous after the heat treatment process compared with base metal. The reason is mainly related to the aggregation of grain boundary precipitates during the heat treatment process. Lots of investigations have pointed out that the difference of intergranular corrosion morphology is related to the distribution and micro-electrochemical properties of grain boundary precipitates [16,18,19]. A higher peak temperature means a longer element diffusion time. Furthermore, the potential difference between matrix and grain boundary increases due to the diffusion of Mg, Zn, and the other active element. Consequently, the intergranular corrosion resistance of the sample after heat treatment at the peak temperature of 350 °C is the lowest among the three samples. In addition, the solute elements of the samples after different heat treatment conditions exist in the matrix with two forms; namely, solid solution and GP zones. The difference in the degree of supersaturation leads to the difference of solute elements’ contents in the matrix. The more the solute content in the matrix, the lower the self-corrosion potential of the matrix, so the tendency to undergo transgranular corrosion increases. Additionally, the difference in the corrosion susceptibility of the samples with different heat treatment conditions is also associated with the value of residual stress, which promotes the extension of corrosion cracking [20].

## 5. Conclusions

The effect of heat treatment peak temperature on the mechanical properties and corrosion behavior of 7N01 aluminum alloy is investigated based on the experiments of micro-hardness, tensile strength, corrosion behavior, and the evolution of microstructure. The main conclusions are as follows:

1. During heat treatment process, most of the initial GP zones dissolve into the matrix, while the grain boundary precipitates have no obvious change. The precipitates of the samples after heat treatment mainly consist of high density GP zones due to the natural aging effect.
2. The mechanical properties of 7N01 aluminum alloy decrease after thermal cycling, while there is no obvious difference in the mechanical properties of the specimens after different heat treatment conditions.
3. The corrosion resistance of heat treatment samples decrease significantly compared with the base metal, which is mainly related to the change of potential difference between the matrix and grain boundary. The recommended heat straightening temperature is 300 °C.

## Figures and Tables

**Table 1:** Chemical composition of 7N01 aluminum alloy (wt.%).

Element	Zn	Mg	Mn	Cr	Zr	Fe	Cu	Ti	Si	V	Al
Content	4.76	1.20	0.42	0.11	0.07	0.11	0.01	0.04	0.04	0.01	93.23

**Table 2:** Evolution of exfoliation corrosion grade with immersion times for 7N01 aluminum alloy in different conditions.

Condition	Immersion Time (h)				
	6	12	24	36	48
Base metal	N	PB	EB	EC	EC
300 °C	N	PC	EB	EC	EC
350 °C	N	PC	EB	EC	EC

Notes: No serious corrosion N; pitting PA (slight pitting in surface) against PC (severe pitting on surface) (with PA weak and PC strong pitting); exfoliation EA (apparent surface layering) to ED (Surface stratification is more serious) (with EA weak and ED strong exfoliation).

**Table 3:** Electrochemistry corrosion parameters of 7N01 aluminum alloy in a 1 M NaCl aqueous solution.

Condition	Corrosion Potential (mV)	Corrosion Current Density (mA/cm^2^)
Base metal	−899 ± 3	(2.15 ± 0.02) × 10^−6^
300 °C	−905 ± 2	(1.93 ± 0.03) × 10^−6^
350 °C	−909 ± 3	(2.09 ± 0.05) × 10^−6^
